# The Role of Age-Related Declines in Subcortical Auditory Processing in Speech Perception in Noise

**DOI:** 10.1007/s10162-016-0564-x

**Published:** 2016-05-23

**Authors:** Tim Schoof, Stuart Rosen

**Affiliations:** 1 UCL Speech, Hearing and Phonetic Sciences, 2 Wakefield Street, London, WC1N 1PF UK; 2 Department of Communication Sciences and Disorders, Northwestern University, Frances Searle Building, 2240 Campus Drive, Evanston, IL 60208 USA

**Keywords:** auditory brainstem response, speech perception, aging, envelope following response, frequency following response

## Abstract

Older adults, even those without hearing impairment, often experience increased difficulties understanding speech in the presence of background noise. This study examined the role of age-related declines in subcortical auditory processing in the perception of speech in different types of background noise. Participants included normal-hearing young (19 – 29 years) and older (60 – 72 years) adults. Normal hearing was defined as pure-tone thresholds of 25 dB HL or better at octave frequencies from 0.25 to 4 kHz in both ears and at 6 kHz in at least one ear. Speech reception thresholds (SRTs) to sentences were measured in steady-state (SS) and 10-Hz amplitude-modulated (AM) speech-shaped noise, as well as two-talker babble. In addition, click-evoked auditory brainstem responses (ABRs) and envelope following responses (EFRs) in response to the vowel /ɑ/ in quiet, SS, and AM noise were measured. Of primary interest was the relationship between the SRTs and EFRs. SRTs were significantly higher (i.e., worse) by about 1.5 dB for older adults in two-talker babble but not in AM and SS noise. In addition, the EFRs of the older adults were less robust compared to the younger participants in quiet, AM, and SS noise. Both young and older adults showed a “neural masking release,” indicated by a more robust EFR at the trough compared to the peak of the AM masker. The amount of neural masking release did not differ between the two age groups. Variability in SRTs was best accounted for by audiometric thresholds (pure-tone average across 0.5–4 kHz) and not by the EFR in quiet or noise. Aging is thus associated with a degradation of the EFR, both in quiet and noise. However, these declines in subcortical neural speech encoding are not necessarily associated with impaired perception of speech in noise, as measured by the SRT, in normal-hearing older adults.

## INTRODUCTION

Older adults typically experience increased difficulties understanding speech in noisy environments, even in the absence of hearing impairment (Dubno et al. 2002; Helfer and Freyman 2008). This has often been attributed to an age-related decline in auditory temporal processing (e.g., Frisina and Frisina 1997; CHABA 1988; Pichora-Fuller and Souza 2003; Pichora-Fuller et al. 2007). Normal-hearing older adults perform more poorly on behavioral measures of temporal processing (e.g., Snell 1997; He et al. 2007). Similarly, neurophysiological studies have shown age-related declines in the temporal precision of subcortical neural sound encoding (e.g., Burkard and Sims 2001; Purcell et al. 2004; Anderson et al. 2012). While the focus of this paper is on the role of age-related declines in subcortical auditory processing, it should be noted that cognitive declines associated with aging may also impact on the ability to understand speech in noise (e.g., Akeroyd 2008; Füllgrabe et al. 2015; but see Schoof and Rosen 2014).

The temporal precision, or fidelity, of subcortical neural coding of complex sounds such as speech is perhaps best assessed by measuring the scalp-recorded frequency following response (FFR) which reflects sustained synchronous neural firing in the brainstem in response to periodic auditory stimuli (Worden and Marsh 1968; Moushegian et al. 1973). Adding FFRs recorded to stimuli of positive and negative polarities is thought to eliminate the cochlear microphonic and any linear stimulus artifacts, and accentuate the envelope of the FFR at its fundamental frequency (F0; Gorga et al. 1985). Subtracting opposite polarity responses, on the other hand, is assumed to reflect phase-locked activity to the temporal fine structure (TFS; Aiken and Picton 2008). However, because envelope cues can be reconstructed from TFS information at the output of peripheral auditory filters, it is difficult to determine to what extent the subtracted polarity FFR indeed reflects TFS information in the stimulus (Ghitza 2001; Heinz and Swaminathan 2009). The focus in this paper is therefore on the added polarity FFR, henceforth referred to as the envelope following response (EFR).

Several studies have shown that EFRs and FFRs are less robust for older compared to younger adults (e.g., Clinard et al. 2010; Vander Werff and Burns 2011; Parbery-Clark et al. 2012; Anderson et al. 2012; Clinard and Tremblay 2013; Marmel et al. 2013). Age-related changes in subcortical processing have been shown, for example, in response to the syllable /dɑ/ (Vander Werff and Burns 2011; Anderson et al. 2012; Clinard and Tremblay 2013). Both Vander Werff and Burns (2011) and Clinard and Tremblay (2013) only found group differences for peaks at the onset and offset of the response. Similarly, Anderson et al. (2012) found increased peak latencies for the older adults only for the onset and formant transition parts of the response and not the steady-state vowel part. However, they also found that several other response measures, such as the response-to-response correlation, phase-locking factor, and rms amplitude, showed age effects both for the transition and steady-state portions of the response. Similarly, age-related changes in subcortical processing have been shown in response to pure tones (Clinard et al. 2010; Clinard and Tremblay 2013; Marmel et al. 2013). Marmel et al. (2013), for example, measured FFRs to pure tones at various frequencies for participants with a wide range of ages and audiometric thresholds. They found that age was significantly correlated with the FFR even when accounting for individual differences in audiometric thresholds.

Previous literature has indicated a relationship between speech perception in noise and the EFR within a group of older adults (Anderson et al. 2011, 2013). Anderson et al. (2011), for instance, showed that older adults with more robust EFRs performed better on a speech-in-noise task than older adults with less robust EFRs. However, while within-group differences in speech-in-noise performance may be attributed in part to differences in subcortical auditory processing, it remains unclear whether the EFR can similarly predict differences in speech-in-noise performance between young and older listeners.

Another question that remains open is whether the EFR can predict the benefit a listener derives when perceiving speech in the presence of a fluctuating compared to a steady-state masker (the fluctuating masker benefit, FMB; Cooke 2006). When a masker fluctuates in amplitude over time, it can be expected that the degrading effect of the noise on the EFR will also vary over time. The degree of “neural masking release” at the troughs of the fluctuating masker may relate to listeners’ abilities to listen in the dips of fluctuating maskers.

The primary aim of this study was to determine the role of age-related declines in subcortical auditory processing in the perception of speech in different types of background noise. Speech perception abilities were assessed in steady-state and amplitude-modulated speech-shaped noise, as well as two-talker babble. In addition, click auditory brainstem responses (ABRs) and EFRs in response to a vowel /ɑ/ in quiet, steady-state, and amplitude-modulated speech-shaped noise were measured.

## METHODS

This experiment was part of a larger study that looked at the relative contribution of age-related declines in both low-level auditory processing and higher level cognitive processing to difficulties in understanding speech in noise typically experienced by older adults (Schoof and Rosen 2014).

### Participants

**FIG. 1:**
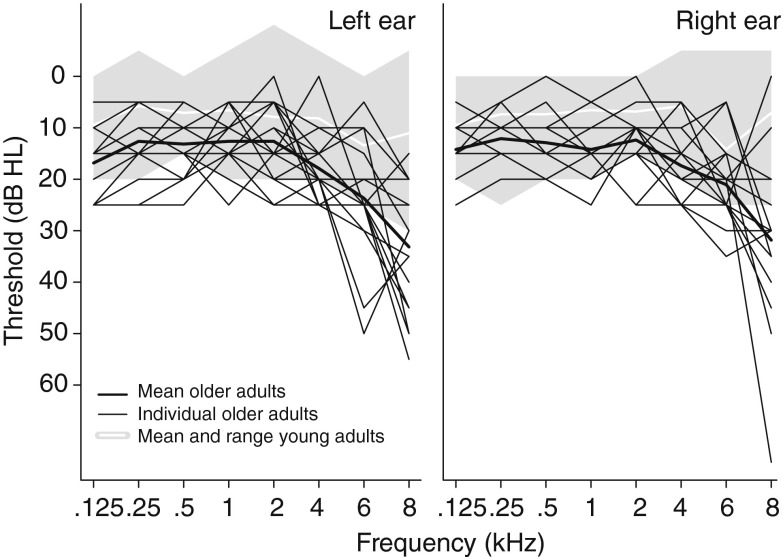
Individual audiograms for older adults are plotted for the left and right ear separately. The *shaded area* represents the range of audiometric thresholds for the younger adults.

Nineteen young (19–29 years old, mean 23.7 years, SD 2.9 years, 10 males) and 19 older (60–72 years old, mean 64.1 years, SD 3.3 years, 3 males) monolingual native English speakers participated in this study. All participants had near-normal hearing defined as (air-conducted) pure-tone thresholds of 25 dB HL or better at octave frequencies from 0.25 to 4 kHz in both ears and at 6 kHz in at least one ear (Fig. 1). Audiometric thresholds were obtained using TDH-49 supra-aural earphones, and the pure tones were presented using a bracketing procedure (down 10 dB, up 5 dB). In addition, all participants over the age of 65 had normal cognitive function (scores >17 MMSE telephone version; Roccaforte et al. 1992). None of the participants reported a history of language or neurological disorders. Participants signed a consent form approved by the UCL Research Ethics Committee and were paid for their participation.

### Speech Perception in Noise

Speech reception thresholds (SRTs) were measured for sentences in different types of background noise. The target stimuli were prerecorded IEEE sentences (Rothauser et al. 1969) produced by a male talker of standard Southern British English. The sentences contained five keywords each and were presented in steady-state speech-shaped noise (SS), speech-shaped noise sinusoidally amplitude-modulated at 10 Hz (AM) with a modulation depth of 100 %, and two-talker babble (see Rosen et al. (2013) for a description of the speech-shaped noise and two-talker babble). The masker started 600 ms before the target sentence and was tapered on and off across 100 ms.

To rule out possible contributions of decreased audiometric thresholds above 6 kHz, the stimuli were low-pass filtered at 6 kHz using a 4th-order Butterworth filter. In addition, for six older participants with thresholds >25 dB HL at 6 kHz in one ear, the stimuli in the relevant ear were spectrally shaped using the National Acoustics Laboratories-Revised (NAL-R) linear prescriptive formula based on their individual thresholds (Byrne and Dillon 1986).

The participants were seated in a soundproof booth and the stimuli were presented binaurally over Sennheiser HD 25 headphones at an overall level of 70 dB SPL. The participants were asked to repeat the sentences as best as they could. The experimenter scored the participants’ responses using a graphical user interface which showed the five keywords. The participants did not receive any feedback.

The signal-to-noise ratio (SNR) was varied adaptively, by fixing the level of the noise and varying the level of the target, following the procedure described by Plomp and Mimpen (1979). Note that the overall stimulus levels were equated for the different SNRs and masker types. The first sentence was presented at an SNR of −10 dB. The initial sentence was repeated and the SNR was increased by 6 dB until at least three out of five keywords were correctly repeated or the SNR reached 30 dB. For each subsequent sentence, the SNR increased by 2 dB for 0–2 correctly repeated keywords or decreased by 2 dB for 3–5 correct repetitions. SRTs were thus tracked at 50 % correct. The number of trials was fixed at 20. The SRT was computed by taking the mean SNR (dB) across the track reversals at the final step size of 2 dB.

SRTs for each condition were measured twice. A measurement was repeated when fewer than three reversals were obtained or when the standard deviation across the final reversals was more than 4 dB.

Participants were familiarized with the task and the different types of background noise. Practice consisted of five sentences with an initial SNR of 0 dB. The order of conditions for the experiment proper was counterbalanced across participants using a Latin square design.

### Electrophysiological Measures

#### Stimuli

Click ABRs were recorded in response to 2000 presentations of a 100-μs click with alternating polarity presented monaurally (left and right separately). Stimuli were presented at 70 dB nHL (107.6 dB peSPL) with a repetition rate of 11/s (Campbell et al. 1981). To confirm the reliability of the measures, two click ABRs were measured for each ear at the start of the session.

EFRs were recorded in response to a synthetic vowel /ɑ/, which was created in MATLAB. The vowel had a fundamental frequency (F0) of 160 Hz (F1: 710, F2: 1200, F3: 2900, F4: 3400 Hz) and a duration of 100 ms. The vowel was tapered on and off across 6.25 ms, which corresponds to one cycle of the F0. EFRs were recorded in response to 3000 presentations of the stimulus in positive and negative polarities separately (i.e. sequentially, not alternating). Stimuli were presented binaurally at 80 dB SPL (measured across the vowel, not including the interstimulus interval) with a repetition rate of 5/s, corresponding to an interstimulus interval of 100 ms. To minimize contamination by stimulus artifact and the cochlear microphonic, the averaged responses to positive and negative polarities were later added together, thus obtaining the EFR (Gorga et al. 1985).

**FIG. 2:**
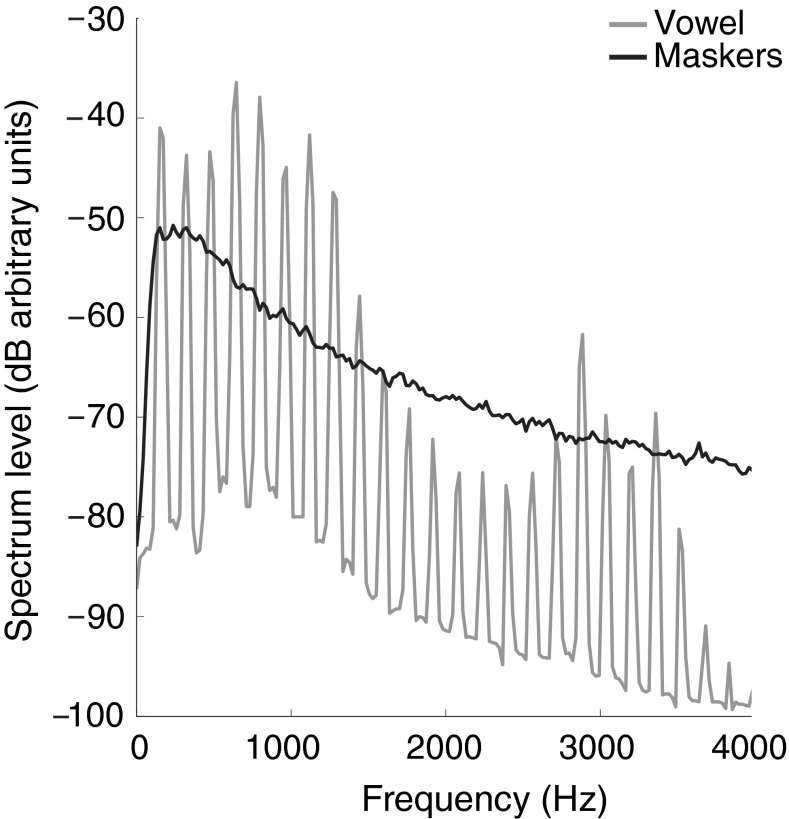
Long-term average power spectra of the vowel /ɑ/ (*gray*) and the two maskers (*black*). The spectral power of the AM masker is equal to that of the SS masker when measured across the 37.5-ms window centered at the peak of the AM masker.

**FIG. 3:**
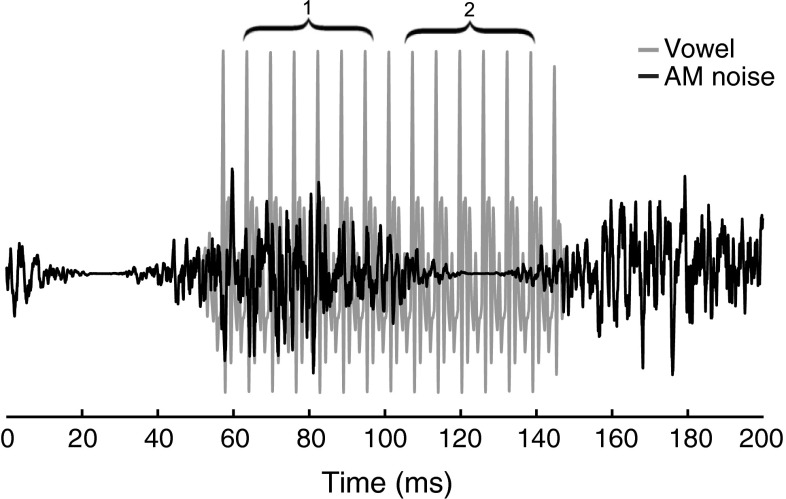
The exact position of the vowel /ɑ/, plotted in *gray*, with respect to the amplitude modulations of the amplitude-modulated speech-shaped noise (AM), plotted in *black*. The vowel starts at 50 ms and spans a whole AM cycle. The two analysis windows, indicated by *curly brackets*, have a duration of 37.5 ms (i.e., 6 F0 cycles). The first window is centered at the peak of the AM noise and the second at the trough of the AM noise. Since the vowel is tapered on and off across 6.25 ms (i.e., 1 F0 cycle), the first analysis window starts 6.25 ms after stimulus onset and the second analysis window ends 6.25 ms before stimulus offset.

EFRs were measured to the vowel in quiet, steady-state speech-shaped noise (SS), and amplitude-modulated speech-shaped noise (AM). Power spectra of the vowel /ɑ/ and the two maskers are plotted in Figure 2. The SS and AM maskers were identical to those used in the SRT task described above. The noise was presented continuously for the duration of the condition and had a rise and fall time of 100 ms. The string of stimuli started playing 225 ms after the start of the masker. Each presentation of the vowel was preceded by a 50-ms prestimulus interval. In the AM condition, the prestimulus period was always centered at the trough of the AM masker (Fig. 3). The SNR was fixed at 7 dB for the SS condition. The level of the AM masker was scaled to give an SNR of 7 dB across a 37.5-ms window centered at the peak of the masker, which corresponds to an SNR of 9.3 dB across the total duration of the AM masker. The SNR for the EFR stimuli was considerably higher (i.e., more favorable) than the SRTs for the speech-in-noise task. However, an SNR similar to the SRTs obtained in the speech tasks would completely drown out the EFR for both young and older participants (c.f. Song et al. 2006; Anderson et al. 2011). Similarly, presenting the stimuli in the speech-in-noise task at more favorable SNRs would result in performance at ceiling.

EFRs in response to each condition were measured twice. The order of conditions (i.e., quiet, SS, AM) was counterbalanced across participants following a Latin square design.

#### Recording Parameters

Participants were seated in a reclining chair in an electrically shielded soundproof booth. To promote stillness, participants were asked to close their eyes and told they were allowed to fall asleep.

Electrophysiological responses were collected using a BioSemi ActiveTwo system (Amsterdam, The Netherlands). Click ABRs were recorded differentially between Cz and the ipsilateral earlobe. EFRs were collected differentially between Cz and the seventh cervical vertebra (C7). Two additional electrodes, Common Mode Sense (CMS) and Driven Right Leg (DRL), were placed near Pz. In the BioSemi ActiveTwo system, these two electrodes replace the ground electrode. The BioSemi system furthermore uses active electrodes (i.e., electrodes that contain preamplifiers), which means that high electrode impedances are tolerated. Instead of minimizing impedances, electrode offsets (i.e., the DC offsets) were minimized. Electrode offsets were always <40 mV. Responses were recorded with a sampling rate of 16,384 Hz.

Stimuli were generated in MATLAB (Mathworks, Natick, MA). The MATLAB script created a series of the required number of stimuli on one channel and a string of triggers on another channel. Both channels were delivered via a computer using an external soundcard (RME FireFace UC, 44.1 kHz) connected to a custom-made trigger box which separated the two channels and simultaneously sent the trigger to the BioSemi machine and the stimulus to electrically shielded ER-3 insert earphones (Intelligent Hearing Systems, Miami, FL). This procedure minimized jitter in the trigger times relative to stimulus presentation. If the presentation of the stimulus and the recording of the response are not precisely time locked, and the EFR is subject to even a small amount of jitter, the EFR would be degraded when sweeps are averaged.

#### Preprocessing

The click ABRs were filtered from 0.1 to 3.0 kHz (2^nd^-order Butterworth filters, going forwards and backwards, therefore zero phase shift) and epoched from −14 to 14 ms relative to the click onset. EFRs were filtered from 0.07 to 2.0 kHz (2^nd^-order Butterworth filters, going forwards and backwards, therefore zero phase shift) and epoched from −49 to 149 ms. Baseline correction was performed with respect to the prestimulus response (−49 to 0 ms). Any epochs containing activity exceeding ±25 μV were rejected.

EFRs were summed across the two runs. This was justified by the fact that they always showed comparable stimulus-to-response correlations and stimulus-to-response lags. The resulting averages (for two runs of 3000 stimulus presentations per polarity) contained, on average, 10,408 sweeps (i.e., epochs; SD 1799). The large variation in the total number of sweeps is mainly due to the fact that responses could not always be summed across the two runs because of missing or noisy data (i.e., containing a number of sweeps with activity exceeding ±25 μV). The EFRs in quiet contained significantly fewer sweeps (i.e., more sweeps were rejected) compared to the EFRs in SS and AM noise (in both groups). However, despite the fact that the EFRs in quiet contained fewer sweeps, they were more robust than the EFRs in SS and AM noise. The number of sweeps did not differ significantly across groups.

#### EFR Analysis

Analyses were performed on the whole EFR as well as across two shorter analysis windows. These two shorter analysis windows were of particular interest for the AM condition to assess encoding of the vowel at the peak and trough of the masker. The windows had a duration of 37.5 ms and encompassed exactly 6 F0 cycles of the vowel (Fig. 3). Given that the stimulus was tapered on and off across 6.25 ms, the responses to the first and last cycles of the vowel were not taken into account in the analyses.

The onset of the EFR was determined objectively by correlating the stimulus (in quiet) with the response (in quiet, SS, and AM noise; Galbraith and Brown 1990). The stimulus waveform was first band-pass filtered at 0.07–2.0 kHz and resampled at 16,384 Hz to match the response waveform. Correlation coefficients were determined by shifting the response relative to the stimulus by 3–10 ms, in 0.06 ms steps, to find the maximum correlation within this time window. The EFR onset was determined for each individual response and used to determine the time window across which to compute subsequent analyses.

The SNR of the response was determined by dividing the root mean square (rms) amplitude of the response to the vowel by the rms of the prestimulus response. Note that the prestimulus response in the AM condition is centered at the trough of the AM cycle (Fig. 3).

#### Assessing the Effects of the Maskers

Nine response properties, computed across the entire EFR, were compared across the three conditions (quiet, SS, AM). Spectral amplitudes were calculated using a fast Fourier transform across 10-Hz wide bins centered at F0 (160 Hz), H2 (320 Hz), and H3 (480 Hz) and taking the peak amplitude within the respective bins. Spectral noise floors were computed by taking the mean spectral amplitude across two 40-Hz wide bins on either side of the spectral components of interest (i.e., F0, H2, and H3). The bins were separated from the relevant spectral component by 5 Hz. The spectral noise floor was calculated across the EFR in response to the stimulus, not the prestimulus baseline. For the spectral analyses, zero-padding to the sampling rate was applied symmetrically around the response to obtain an FFT with a resolution of 1 Hz, thus ensuring that the spectral components of the EFR fell at the center of a bin. Values reported here are peak amplitudes of the power spectrum, in dB relative to 1 μV rms. The rms amplitude of the EFR (in dB relative to 1 μV rms) was computed as an indication of the magnitude of the response. The encoding of pitch information was quantified using an autocorrelation function across a 40-Hz wide analysis window centered at the F0 of the stimulus (160 Hz). The height of the first peak in the autocorrelation function provided a measure of pitch strength (c.f. Krishnan et al. 2005). Stimulus-to-response lags and correlations were computed to provide an overall measure of the robustness of encoding. Cross-correlation coefficients were computed across responses of different conditions to assess the effect of the noise on the robustness of encoding of the vowel. Cross-correlations were computed for quiet to SS, quiet to AM, and AM to SS. Responses were shifted relative to one another across −6 to +6 ms, in 0.06 ms steps. Similarly, cross-correlations were calculated for responses of the same condition across the two different runs. A Fisher transformation was used to convert the correlation coefficients (*r* values) to z-scores for statistical analyses.

#### Assessing the Effect of Amplitude Modulations in the Masker

To examine the effect of the modulations in the AM masker, spectral amplitudes at F0 (160 Hz), H2 (320 Hz), and H3 (480 Hz); pitch strength; and rms amplitude were compared across the two analysis windows.

## RESULTS

EFR data from one older participant was excluded from the analyses due to a potential stimulus artifact in the recordings. While the stimulus artifact may not have been a problem for the EFRs, as adding polarities typically removes any linear stimulus artifacts, the responses for this participant were excluded to be on the safe side. The artifact was visually identified in the subtracted polarity response (but not the added polarity EFR). The timing and magnitude of the response indicated that it could not have originated from the brainstem. Extensive testing was done before the start of the study to rule out any artifacts by recording responses, while participants’ ears were plugged so the stimuli were inaudible. No artifacts were detected in this testing period. It is unclear why there may have been a stimulus artifact for this particular older adult.

Furthermore, except where stated otherwise, outliers were excluded in the analyses reported below if they exceeded the mean ± 3 SD. In total, 19 data points, which were spread randomly across participants and EFR measures were excluded (EFR quiet: one young and one older adult for stimulus-to-response lag, F0, H2, H3, SNR; EFR SS: one young adult for H3, four young and two older adults for response-to-response correlation; EFR AM: one young and one older adult for response-to-response correlation).

### Speech Perception in Noise

**FIG. 4:**
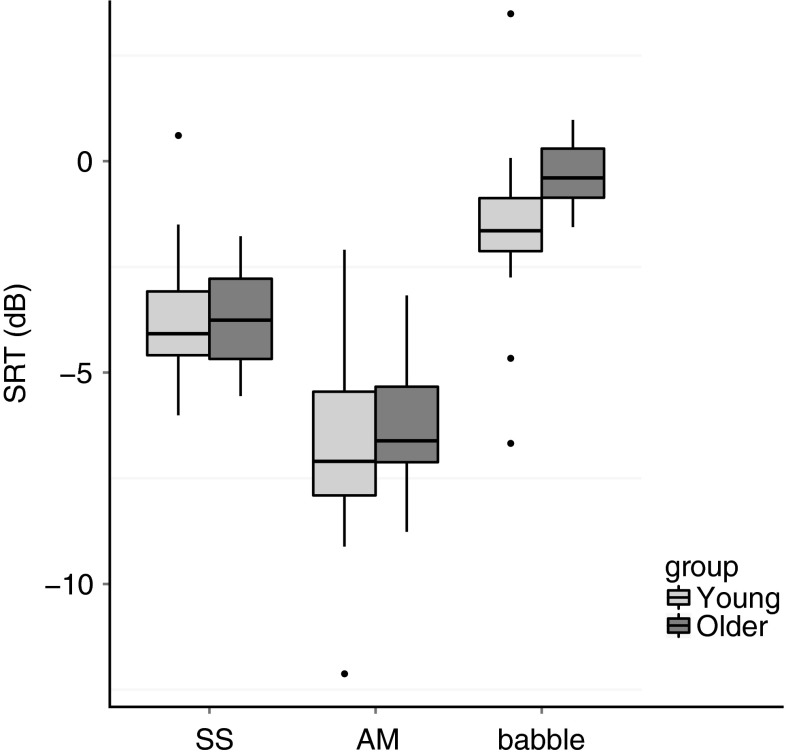
Boxplots of speech reception thresholds (SRT, in dB SNR) for young (*light gray*) and older (*dark gray*) listeners for SS noise (*left*), AM noise (*middle*), and two-talker babble (*right*). The *boxes* show the interquartile range (25th–75th percentile) of the data with the *horizontal line* indicating the median (50th percentile). The whiskers indicate values that fall within 1.5 times the interquartile range, and values falling outside that range are indicated by a *dot*.

The results of the speech-in-noise task are displayed in Figure 4. The figure shows SRTs for both young and older groups in SS noise, AM noise, and two-talker babble.

Older adults were expected to have higher (i.e., worse) SRTs in the presence of all three maskers (SS, AM, two-talker babble). As discussed in Schoof and Rosen (2014), however, the older adults only performed worse in the presence of two-talker babble. A mixed-effects model with condition (SS, AM, babble) and group (young, old) as fixed factors and participant and sentence list as random factors showed a significant interaction between group and condition [*F*_(2, 186)_ = 5.6, *p* = 0.004]. Post hoc independent *t* tests revealed a significant difference between the two age groups for SRTs in babble only, with young listeners performing on average 1.4 dB better (i.e., lower SRTs) than older listeners [*t*_(36)_ = 2.8, *p* = 0.008, Cohen’s *d* = 0.9; all other *p* > 0.6].

Figure 4 illustrates that both groups showed a dip listening effect as indicated by lower (i.e., better) SRTs in AM than SS noise. In addition, SRTs in babble were higher (i.e., worse) compared to the two noise maskers. These findings were supported by independent *t* tests. SRTs in AM were on average 2.7 dB lower than in SS noise [*t*_(37)_ = 12.9, *p* < 0.001, Cohen’s *d* = 1.4], indicative of dip listening. In addition, SRTs in babble were significantly higher than in SS and AM noise by 2.6 and 5.3 dB, respectively [SS: *t*_(37)_ = 8.5, *p* < 0.001, Cohen’s *d* = 2.5; AM: *t*_(37)_ = 16.3, *p* < 0.001, Cohen’s *d* = 1.4].

### Subcortical Auditory Processing

#### Click ABRs

To assess whether any age-related changes in subcortical auditory processing were evident in the click ABR, wave V peak latencies and amplitudes of the response were compared between young and older adults. Independent *t* tests indicated that the responses were not significantly affected by age [latency *t*_(33)_ = −0.58, *p* = 0.57, amplitude *t*_(34)_ = −1.37, *p* = 0.18]. It should be noted that responses for one young (one ear) and three older adults (both ears for one of the three older adults) were abnormal, defined as having latencies larger than three standard deviations above the mean for the group of young adults (>6.54 ms; c.f. Campbell et al. 1981). These participants were not excluded from the analyses because their ABRs were repeatable.

#### EFRs

**FIG. 5:**
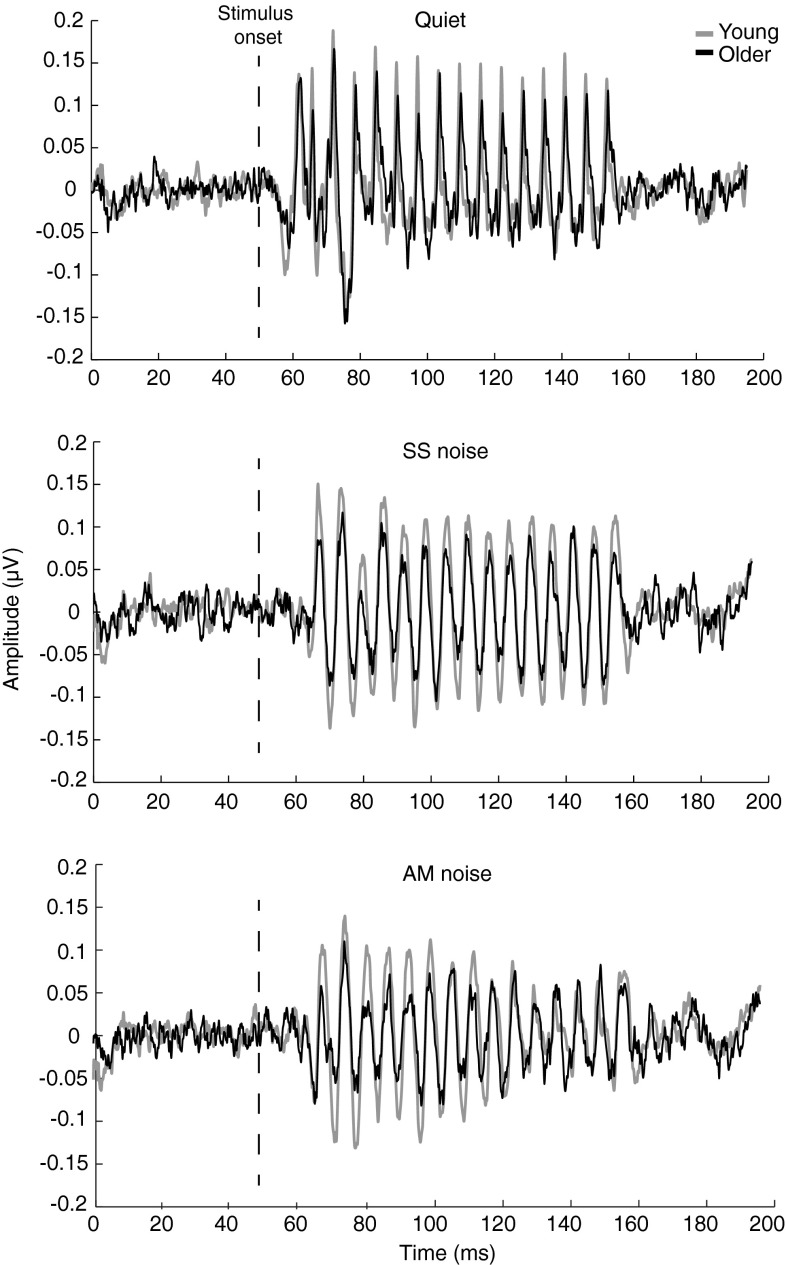
Grand averaged EFRs in quiet (*top*), SS noise (*middle*), and AM noise (*bottom*) for young (*gray*) and older (*black*) listeners. The *dashed line* marks the stimulus onset (i.e., the vowel).

**FIG. 6:**
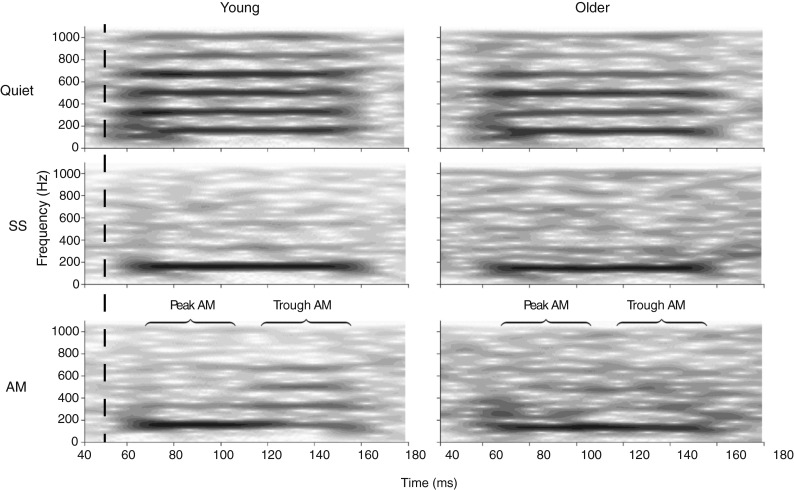
Spectrograms of grand averaged EFRs in quiet (*top*), SS noise (*middle*), and AM noise (*bottom*) for young (*left*) and older (*right*) listeners. The *dashed line* marks the stimulus onset (i.e., the vowel).

Grand averaged responses for the two age groups in quiet, SS, and AM noise are plotted in Figure 5. The figure illustrates that the EFRs in all three conditions are less robust for the group of older adults. Spectrograms of the grand averaged EFRs are shown in Figure 6, which illustrates the effects of background noise on the response. In SS noise, for example, the spectral component at F0 is preserved while the spectral magnitudes of the harmonics are greatly reduced. Furthermore, the effects of the amplitude modulations of the AM masker are evident in that the spectral magnitudes of the harmonics at the peak of the masker are greatly reduced (as in SS noise) compared to the trough of the masker (as in quiet). The age-related declines in the EFR are perhaps less pronounced in this figure. Instead, it may appear as if the difference in EFRs is the result of increased “neural noise” (i.e., spontaneous brain activity) in the older group (Salthouse and Lichty 1985; Bidelman et al. 2014). However, this is not supported by statistical analyses and is likely attributable to the automatic gain control settings of the spectrogram.

While responses with an SNR <1.5 dB are typically excluded from analyses (Skoe and Kraus 2010), this rule of thumb was not applied here since the distribution of SNRs (of the response) was not spread equally across the different groups and conditions. Results from a mixed-effects model for SNR with condition (quiet, SS noise, and AM noise), group (young, old), and number of sweeps as fixed factors and participant as a random factor revealed a significant effect of condition [*F*_(2, 60)_ = 4.6, *p* = 0.01] and group [*F*_(1, 35)_ = 7.5, *p* = 0.01] but no condition × group interaction [*F*_(2, 60)_ = 0.5, *p* = 0.6] or an effect of number of sweeps [*F*_(1, 60)_ = 1.4, *p* = 0.2]. Closer examination revealed that SNRs were higher in young compared to older adults, and a planned contrast (quiet vs. AM and SS) showed that the SNRs in both SS and AM noise were significantly lower than in quiet [*t*_(60)_ = 2.8, *p* = 0.006]. However, SNRs for the two noise conditions did not differ significantly [*t*_(60)_ = 0.6, *p* = 0.5].

To assess whether the group difference in SNR was the result of increased “neural noise,” that is, spontaneous brain activity (e.g., Salthouse and Lichty 1985; Bidelman et al. 2014), a mixed-effects model with condition (quiet, SS noise, and AM noise) and group (young, old) as fixed factors and participant as a random factor was conducted on the rms amplitude of the prestimulus baseline. The analysis did not reveal a significant main effect of group [*F*_(1, 35)_ = 1.2, *p* = 0.3] or a significant interaction between group and condition [*F*_(2, 68)_ = 0.02, *p* = 0.97]. This suggests that the group difference in SNR is not attributable to increased “neural noise” in the older group. The group difference in SNR is therefore most likely the result of a reduction in the EFR in response to the stimulus in the older group.

The question of whether the EFRs for the older adults showed evidence of increased “neural noise” was also examined in the frequency domain. A mixed-effects model with condition (quiet, SS noise, and AM noise), group (young, old), frequency (F0, H2, and H3), and number of sweeps as fixed factors and participant as a random factor was conducted on the spectral noise floor (calculated across the EFR in response to the stimulus, not the prestimulus baseline). The analysis revealed no significant main effect of group [*F*_(1, 35)_ = 2.2, *p* = 0.1] nor significant interactions with group and condition and/or frequency [all *p* > 0.4].

#### Effects of Aging and Noise Maskers on the EFR

A large number of response properties were computed on the EFRs (nine to assess the effects of background noise and five to examine the “neural masking release”), but examining the effects of group and condition on all these individual EFR measures would have led to very stringent statistical criteria after Bonferroni correction. The same applies to calculating correlations with all of the individual EFR measures with the SRTs to examine the relationship between subcortical auditory processing and speech-in-noise performance. On the other hand, selecting a small number of measures out of the plethora available is also difficult as we do not know which are relevant, given that the underlying processes responsible for many, if not all, of these measures are not clearly understood. We argue that it is more insightful to look at a small number of overall measures of subcortical speech encoding and relate these to speech-in-noise performance. These overall EFR measures were computed by means of principal components analysis (PCA). For detailed analyses of the effects of group (young, old) and condition (quiet, AM, SS noise) on the individual EFR measures, see the Appendix.

PCA is a data reduction technique that transforms a set of correlated variables into a smaller set of uncorrelated variables, called principal components (Pearson 1901; Hotelling 1933). These principal components reflect linear combinations of the input variables that account for the largest proportion of variance in the data set. While PCA is a widely used statistical technique, it has not yet been applied to EFRs or FFRs. We argue that EFRs and FFRs lend themselves perfectly to PCA because they typically consist of a large set of correlated measures (think for example of spectral magnitude at F0 and the harmonics).

Three missing data points (i.e., 19 data points that were considered outliers and therefore excluded) were first imputed using a regression technique with an estimation adjustment based on the residuals. Subsequently, PCA was performed on all EFR measures using varimax rotation with Kaiser normalization. The resulting principal components (PCs) were saved as Anderson-Rubin scores, which ensures the PC scores are uncorrelated.

Seven principal components with eigenvalues >1 were initially extracted. However, the majority of these PCs explained less than 10 % of the variance in the data. Therefore, percentage of variance explained by each individual component (≥10 %) was used as an additional guiding principle for the number of PCs to be extracted. It should be noted that this guiding principle was applied to the *unrotated* components since the percentage of variance explained by rotated components changes depending on the number of components that are extracted. Three (rotated) components were finally extracted. Together they explained 62 % of the variance in the data, with PC1 accounting for 30 %, PC2 for 19 %, and PC3 for 13 % of the variance (percentages refer to the variance explained by the rotated components; rotated factor loadings for these components are shown in Table 1).

**TABLE 1:** Rotated components matrix showing the three extracted principal components for the individual EFR measures on responses in quiet, SS, and AM noise

Rotated components matrix—EFR				
Condition	Measure	PC1	PC2	PC3
Quiet	Stimulus-to-response correlation	−0.04	0.32	−0.13
Quiet	Stimulus-to-response lag	−0.07	**0.51**	0.24
Quiet	Spectral power F0	−0.29	**0.81**	0.02
Quiet	Spectral power H2	0.24	**0.60**	**0.52**
Quiet	Spectral power H3	0.17	**0.71**	**0.47**
Quiet	Rms amplitude	−0.05	**0.85**	0.32
Quiet	Response-to-response correlation	0.22	**0.89**	−0.02
Quiet	Pitch strength	**0.79**	0.12	0.05
Quiet	SNR	0.27	**0.91**	0.08
SS	Stimulus-to-response correlation	**0.72**	0.20	−0.33
SS	Stimulus-to-response lag	−0.22	−0.11	**0.84**
SS	Spectral power F0	**0.89**	−0.04	−0.06
SS	Spectral power H2	0.27	−0.11	**0.55**
SS	Spectral power H3	0.05	0.06	**0.50**
SS	Rms amplitude	**0.73**	−0.18	0.25
SS	Response-to-response correlation	**0.84**	0.19	−0.27
SS	Pitch strength	**0.85**	0.16	0.10
SS	SNR	**0.82**	−0.07	0.00
AM	Stimulus-to-response correlation	**0.75**	0.07	−0.13
AM	Stimulus-to-response lag	−0.14	0.16	**0.72**
AM	Spectral power F0	**0.85**	0.04	0.20
AM	Spectral power H2	0.16	0.05	0.38
AM	Spectral power H3	−0.06	0.28	**0.53**
AM	Rms amplitude	**0.69**	−0.11	**0.41**
AM	Response-to-response correlation	**0.87**	0.14	0.04
AM	Pitch strength	0.23	**0.82**	−0.15
AM	SNR	**0.77**	−0.04	0.24

Factor loadings >0.4 are highlighted in bold. Rotation of the components matrix maximizes the loading of each EFR measure on one of the extracted principal components and minimizes the loading on all other components. It thus simplifies the interpretation of the factor loadings

**FIG. 7:**
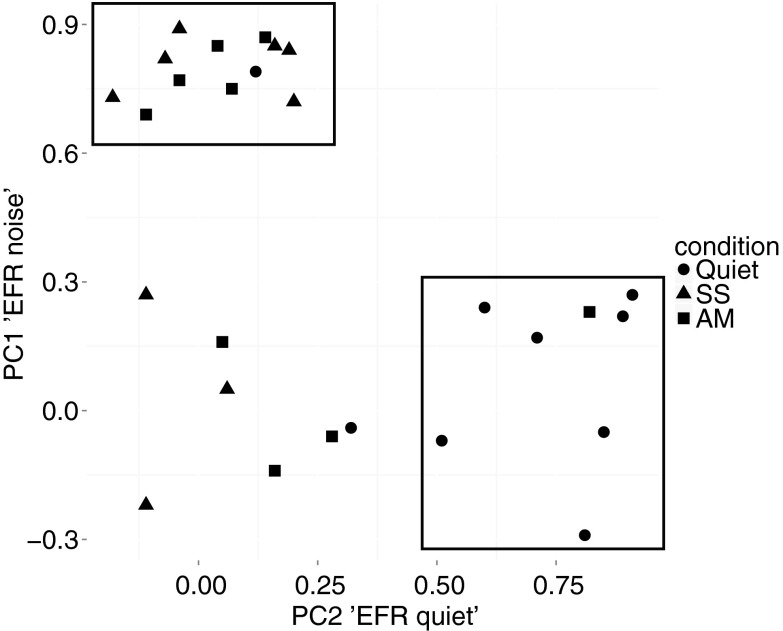
Factor loadings for the individual EFR measures on responses in quiet (*circles*), SS noise (*triangles*), and AM noise (*squares*) onto the two extracted principal components (PC1 “EFR noise” and PC2 “EFR quiet”). The *rectangles* group the EFR measures with factor loadings above 0.4 on one of the principal components. This plot illustrates that the PC1 “EFR noise” is dominated by measures on the EFR in AM and SS noise, while the PC2 “EFR quiet” is dominated by measures on the EFR in quiet.

Figure 7 shows the factor loadings of the first two principal components graphically (c.f. Table 1). The individual data points reflect the factor loadings onto PC1 and PC2 for the 27 different EFR measures (taking together data from both age groups). The figure shows that measures from the EFRs in noise (AM and SS) predominantly load on PC1 and measures from the EFRs in quiet load primarily onto PC2. PC1 was therefore interpreted as reflecting subcortical speech encoding in noise (“EFR noise”), while PC2 was interpreted to reflect EFRs in quiet (“EFR quiet”). The fact that measures of the EFRs to the vowel in the presence of both AM and SS noise loaded onto PC1 suggests that the EFR was affected by background noise, regardless of whether the noise was steady state or fluctuated in amplitude over time.

To examine whether the EFRs were (overall) significantly different across groups, two one-sided independent *t* tests were conducted on the principal components measures. The results showed that the groups differed significantly in terms of these first two components [PC1 “EFR noise” *t*_(35)_ = 2.6, *p* = 0.007; PC2 “EFR quiet” *t*_(35)_ = 2.3, *p* = 0.014; significant after Bonferroni correction], with higher (i.e., better) scores for the younger compared to the older listeners. In other words, the EFRs were more robust for the younger than for the older adults.

The loadings for the third PC were a subset of those of the first two components and were therefore not considered very informative. Moreover, these components did not show a significant difference between the groups [PC3 *t*_(35)_ = −0.1, *p* = 0.5]. Consequently, subsequent analyses will only focus on the first two PCs.

It is important to note that the results of the PCA are in line with the results of the analyses on the individual EFR measures (see Appendix). The individual EFR measures mostly also showed an effect of background noise, irrespective of its type. Similarly, the EFR measures were typically less robust for the older compared to the younger adults. While both analyses give the same result, the advantage of the PC measures is that they reflect an overall measure of the EFR and circumvent the problem of multiple comparisons, especially when relating the electrophysiological to the behavioral data.

As an additional cross-validation, PCAs were performed on the two age groups separately. As before, three components were extracted. The factor loadings of the PCs for both age groups showed the same pattern of results as those for both groups combined reported above. Measures from the EFRs in AM and SS noise loaded predominantly onto PC1 and measures from the EFRs in quiet loaded primarily onto PC2. Moreover, factor loadings for PC1 and PC2, but not PC3, were highly correlated across the two age groups [PC1 *r* = 0.66, *p* < 0.001, PC2 *r* = 0.53, *p* = 0.005, PC3 *r* = 0.06, *p* = 0.8].

Similarly, an overall measure of “neural masking release” (i.e., the difference in EFR response measures in AM noise between the peak and trough of the masker) was computed using PCA. Two components with eigenvalues >1 were extracted. Together these (rotated) components explained 76 % of the variance in the data, with PC1 accounting for 51 % and PC2 accounting for 25 % of the variance (rotated factor loadings are shown in Table 2). The first PC was interpreted as reflecting neural masking release related to the fundamental (PC “neural masking release—F0”; F0, pitch tracking, rms amplitude), and the second PC was interpreted as neural masking release affecting the harmonics (PC “neural masking release—harmonics”; second and third harmonics).

**TABLE 2:** Rotated components matrix showing the two extracted principal components for the EFR difference measures (i.e. response measure at the peak minus the trough of the AM masker), as an indication of “neural masking release”. Factor loadings > 0.4 are highlighted in bold font. Other details as for Table 1

Rotated components matrix neural masking release		
Measure	PC1	PC2
Spectral power F0	**0.925**	−0.178
Spectral power H2	0.341	**0.610**
Spectral power H3	−0.110	**0.863**
Rms amplitude	**0.921**	**0.241**
Pitch strength	**0.844**	0.201

To examine whether the young and older adults differed in terms of “neural masking release,” two independent *t* tests were conducted. The results showed a significant difference between the groups in terms of the principal component reflecting encoding of F0 [*t*_(30)_ = −2.8, *p* = 0.009], with higher scores for the older compared to the younger adults. It should be noted that lower, and not higher, scores on this measure are actually indicative of a larger neural release from masking. This is because, contrary to expectations, the rms and spectral magnitude at F0 were higher in the peak compared to the trough of the AM masker, at least for young adults (see Appendix). Since neural release from masking was calculated by subtracting the values measured at the peak of the AM masker from values measured at the trough, more negative scores reflect a larger masking release. This means that in terms of the F0 principal component measure, the younger adults experienced a larger “neural masking release” compared to the older adults. By contrast, no significant group difference was found for the principal component reflecting the harmonics [*t*_(30)_ = 1, *p* = 0.3].

Again, as a cross-validation of the PCA, the analysis was performed on the two age groups separately as well. The factor loadings of the PCs for both age groups showed the same pattern of results as those for both groups combined reported above, with spectral magnitude at F0, rms amplitude, and pitch strength loading onto PC1 and the second and third harmonics loading onto PC2. No correlation analyses on the factor loadings were conducted as the number of factors (five) was considered too small.

#### Effects of Differences in Audiometric Thresholds on the EFR

While both young and older adults had near-normal hearing, defined as pure-tone thresholds ≤25 dB HL up to 4 kHz in both ears and at 6 kHz in at least one ear, thresholds for the two groups were significantly different. Independent *t* tests indicated that pure-tone averages (PTA) across 0.5–4 kHz (≤25 dB HL) and 6–8 kHz (some ≥25 dB HL) were significantly higher (i.e., worse) for the older age group [500–4000 Hz: *t*_(36)_ = −6.4, *p* < 0.001, mean difference 7 dB; 6–8 kHz: *t*_(36)_ = −8.2, *p* < 0.001, mean difference 16 dB]. This raises the question whether the observed degradations in the EFR were indeed the result of aging or were in fact attributable to differences in audiometric thresholds.

To assess whether the degradation of EFRs in the older adults was driven by an elevation in audiometric thresholds, linear regressions were performed on the two principal component measures of the EFR independently (PC1 “EFR noise” and PC2 “EFR quiet”), with age group entered into the model after accounting for individual differences in PTA across 0.5–8 kHz [PTAs across 0.5–4 and 6–8 kHz were not entered separately since the two measures were highly correlated; *r* = 0.74, *p* < 0.001].

**FIG. 8:**
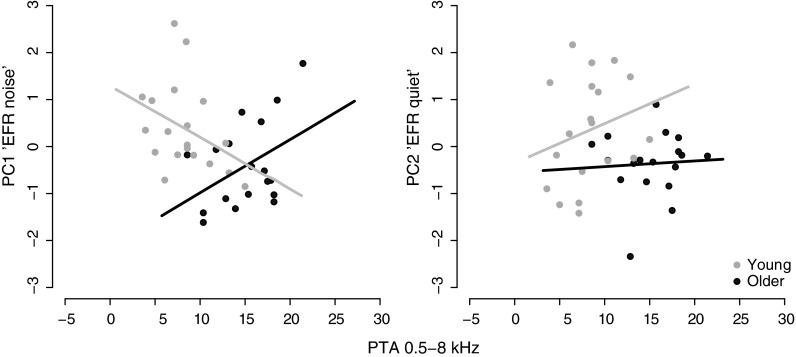
Scatter plots illustrating the relationship between the principal component measures of the EFR (PC1 “EFR noise” and PC2 “EFR quiet”) and audiometric thresholds (pure-tone average across 0.5–8 kHz). Data points for the older adults are in *black* and for the younger adults in *gray*. Best-fitting lines are plotted to illustrate the relationship between the two measures across both groups.

The analyses indicated that individual differences in the EFR in quiet were predicted by age group, even after accounting for differences in audiometric thresholds (see Table 3 and Fig. 8). However, this result was not significant after Bonferroni correction. The regression analysis on the PC “EFR noise” shows that age group did not significantly predict variability in the EFR in noise after accounting for differences in audiometric thresholds. However, audiometric thresholds did not significantly predict the EFR in noise either. It is important to point out that since aging is associated with elevated audiometric thresholds, it is difficult to tease apart the effects of the two variables on subcortical speech encoding. This is supported by the collinearity statistics [Tolerance = 0.5, VIF = 2], which indicate that multicollinearity may be biasing the regression analyses.

**TABLE 3:** Results of the linear regression analyses on the EFR principal components (* significant at α = 0.05, highlighted in bold font). Note that *β* refers to the standardised regression coefficient and SE stands for the standard error of the regression coefficient *b*. The *R* ^2^ reflects the proportion of the variance accounted for as predictors are added to the model. 95 % confidence intervals (CI) for the regression coefficients are also given

		Linear regression					
Measure	Predictors	*b*	*β*	SE	*p*	*R* ^2^	CI
PC1 “EFR noise”	PTA 0.5−8 kHz	0.0	0.04	0.05	0.9	0.07	[−0.09, 0.1]
	Group	−0.4	−0.42	0.23	0.08	0.1	[−0.9, 0.04]
PC2 “EFR quiet”	PTA 0.5–8 kHz	0.05	0.2	0.05	0.4	0.03	[−0.05, 0.14]
	Group	−0.5	−0.52	0.23	0.03*	0.15	[−0.98, −0.005]

### Predicting Speech Perception in Noise

The results thus far suggest that, compared to the young participants, the older adults had no problems understanding speech in SS and AM noise, despite a decline in subcortical auditory processing and a comparative elevation in audiometric thresholds. The question remains, however, whether age-related changes in subcortical auditory processing can account for the group difference in SRTs in babble.

To answer this question, a best subsets regression analysis (Hastie et al. 2009) was performed on the SRTs in babble with age group, PTA across 0.5–4 kHz, and the two principal component measures of the EFR (PC1 “EFR noise” and PC2 “EFR quiet”). The final regression model was selected based on the Bayesian Information Criterion (BIC; Schwarz 1978). The analyses suggested that SRTs in babble were best predicted by PTA across 0.5–4 kHz [*R*^2^ = 0.2, *F*_(1, 35)_ = 9.2, *p* = 0.004, see Table 4]. Thus, age-related declines in subcortical auditory processing did not predict SRTs in babble beyond group differences in audiometric thresholds.

**TABLE 4:** Results from best subsets regression analyses on SRTs in babble, the averaged SRT in AM and SS noise, and the fluctuating masker benefit (FMB). Significant results are highlighted in bold font (* significant at α = 0.05, ** significant at α = 0.01). See the caption for Table 3 for other details

Measure	Predictors	Regression on SRTs					
		*b*	*β*	SE	*p*	*R* ^2^	CI
SRT babble	PTA 0.5–4 kHz	0.14	0.5	0.05	0.004**	0.2	[0.05, 0.25]
SRT noise	PTA 0.5–4 kHz	0.08	0.2	0.05	0.1	0.25	[−0.03, 0.17]
FMB	Wave V latency	−2.3	−0.5	0.78	0.006**	0.11	[−3.9, −0.7]
	Wave V amplitude	−6.6	−0.4	2.5	0.01*	0.16	[−11.8, −1.5]

A question that remains is whether individual differences in audiometric thresholds and/or the subcortical auditory processing can account for the variability in SRTs in SS and AM noise. Since the SRTs in AM and SS noise were highly correlated [*r* = 0.736, *p* < 0.001, *R*^2^ = 0.54], the best subsets regression was performed on the average of the two, with age group, PTA across 0.5–4 kHz, and the two principal component measures of the EFR (PC1 “EFR noise” and PC2 “EFR quiet”) as possible predictors.

The results indicated that a model with PTA across 0.5–4 kHz also best explained the variability in SRTs in the two noise maskers [*R*^2^ = 0.06, *F*_(1, 36)_ = 2.3, *p* = 0.1, see Table 4]. It should be noted, however, that PTA did not in fact significantly predict SRTs in noise. Furthermore, the results show that age-related declines in subcortical auditory processing, as assessed by the EFR to a single vowel, could not predict SRTs in noise.

### Predicting Fluctuating Masker Benefit

The results described above showed that response properties of the EFR tended to be more robust at the trough than at the peak of the AM masker. A question that remains, however, is whether the amount of neural release from masking could predict the amount of FMB a listener derives. It should be noted that the data did not show any age differences in terms of either the amount of neural release from masking or the amount of FMB. However, this does not mean the two measures could not be correlated.

In order to answer the question whether neural release from masking could predict FMB over and above simpler measures such as audiometric thresholds and the click ABR, a best subsets regression was conducted on the FMB with age group, PTA across 0.5–4 kHz, PC “neural masking release—F0,” PC “neural masking release—harmonics,” and click ABR wave V amplitude and latency as possible predictors.

The results revealed that a model with wave V amplitude and latency best explained the variability in FMB [*R*^2^ = 0.27, *F*_(2, 31)_ = 5.7, *p* = 0.008; collinearity statistics Tolerance = 0.8, VIF = 1.2; see Table 4]. Increased wave V latencies, perhaps indicative of prolonged neural recovery times, were associated with smaller FMBs (Fujikawa and Weber 1977; Debruyne 1986; Walton et al. 1999). However, the direction of the relationship between FMB and wave V *amplitude* was opposite to what might be expected, with larger wave V amplitudes associated with smaller FMBs. It should be also noted that the standard error associated with the regression coefficient of wave V amplitude was relatively large (see Table 4). The relationship between the click ABR measures and the FMB is thus not very convincing. It is furthermore important to note that the amount of neural masking release did not predict the FMB.

## DISCUSSION

The primary aim of this study was to determine whether declines in subcortical auditory processing could explain the increased difficulties older adults can experience in the perception of speech in different types of background noise.

In line with previous research, the data revealed an age-related decline in the robustness of subcortical neural speech encoding (c.f. Vander Werff and Burns 2011; Anderson et al. 2012; Clinard and Tremblay 2013). While Vander Werff and Burns (2011) and Clinard and Tremblay (2013) only found age differences at the onset and offset of the EFR, our data reveal an age-related decline in EFR properties in response to the sustained vowel. These findings are in agreement with Anderson et al. (2012) who found age effects not only for the onset and formant transition period but also in response to the sustained vowel portion of responses to the syllable /dɑ/. It is important to stress that the EFR was not more affected by noise in older than younger listeners.

It may be the case that the decline in the robustness of subcortical neural speech encoding in the older group is a result of age-related hearing loss as opposed to aging per se. Even though the older adults meet quite strict criteria for normal hearing (thresholds ≤25 dB HL up to 6 kHz in at least one ear and up to 4 kHz in both ears), their audiometric thresholds were higher compared to the younger adults. While the exact origins of the components of the EFR are not yet entirely clear, it is generally understood that delays imposed by the traveling wave along the basilar membrane play an important role (Don and Eggermont 1978; Dau 2003; Nuttall et al. 2015). Moreover, it has been suggested that phase-locked activity to the stimulus primarily stems from neurons at more basal sites, especially at high intensities (Janssen et al. 1991; Dau 2003). Differences in audiometric thresholds, especially in the higher frequencies, may thus have contributed to a decreased robustness of the EFR in the older adults. It is difficult to say, however, whether the observed group differences in the EFR were primarily the result of elevated audiometric thresholds or aging, since the two go together. Regression analyses indicated that age group significantly predicted the principal component reflecting EFRs in quiet, even after accounting for individual differences in audiometric thresholds (although this was not significant after Bonferroni correction; see also Marmel et al. 2013; Vander Werff and Burns 2011). By contrast, however, variability in the principal component reflecting EFRs in noise could not clearly be predicted by either age group or audiometric threshold.

Another interesting question is whether the decreased robustness of the EFR in the older group is in part due to an increase in spontaneous brain activity, or increased “neural noise” (c.f. Salthouse and Lichty 1985; Bidelman et al. 2014). Bidelman et al. (2014), for example, found increased neural activity for older compared to younger adults during interstimulus intervals when recording FFRs. However, they did not find group differences in resting-state EEG (when no sound was playing), suggesting that the two age groups were not inherently different in terms of spontaneous brain activity. Our data did not show an age-related increase in neural activity during the prestimulus baseline (or interstimulus interval), as indicated by similar rms amplitudes for the two age groups. Similarly, the data did not reveal an age-related increase in the spectral noise floor during stimulus presentation.

It was hypothesized that declines in subcortical auditory processing would lead to increased difficulties understanding speech in noise. However, the data showed that the normal-hearing older adults were in fact fairly unimpaired in their speech-in-noise perception abilities. The older adults only performed more poorly on the speech-in-noise task compared to the younger listeners in the presence of two-talker babble (in line with, e.g., Rajan and Cainer 2008; c.f. Helfer and Freyman 2008). The fact that the older adults performed more poorly in the presence of the competing speech but not the two noise maskers suggests that older adults may be more susceptible to informational masking (c.f. Freyman et al. 2004) or less able to benefit from periodicity cues to segregate the target and masker. Contrary to expectations, the older adults did not show a reduced FMB (Stuart and Phillips 1996; Peters et al. 1998; Dubno et al. 2002, 2003; Gifford et al. 2007; Grose et al. 2009). A possible explanation for this apparent discrepancy with previous literature is that most studies in the past have used simpler target stimuli, such as syllables (e.g., Stuart and Phillips 1996; Dubno et al. 2002, 2003) or simple BKB or HINT sentences (e.g., Gifford et al. 2007), as opposed to the more complex IEEE sentences used in this study (but see Grose et al. 2009). While age-related differences may be expected to increase, rather than decrease, with increased task complexity, in this case, the older adults may have been able to benefit from an increase in vocabulary size associated with aging when trying to understand the more complex IEEE sentences (Verhaeghen 2003). An alternative explanation is that the older adults who participated in our study may have been “super-agers,” evidenced in part by the fact that they had normal or near-normal hearing. Worth noting is that these same older adults showed no age-related declines, as has been found in other aging studies, in behavioral measures of either envelope or TFS processing (Schoof and Rosen 2014), again supporting the notion of “super-agers.” Furthermore, it should be noted that the idea that older adults benefit less from fluctuations in the masker compared to young listeners is perhaps somewhat controversial since age-related reductions in the FMB reported in the literature may in part have been the result of inherent group differences in steady-state background noise (c.f. Stuart and Phillips 1996; Dubno et al. 2002, 2003; Bernstein and Grant 2009).

The results of this study suggest that normal-hearing older adults may be unimpaired in their perception of speech in the presence of AM and SS noise when complex, ecologically valid target stimuli are used, despite declines in subcortical auditory processing. Moreover, the small increase in SRTs in babble could not be explained in terms of the age-related changes in subcortical auditory processing. Instead, individual differences in SRTs in the presence of two-talker babble, although not the noise maskers, were best explained in terms of differences in audiometric thresholds across 0.5–4 kHz. This suggests that declines in the precision of temporal neural coding do not necessarily lead to increased perceptual difficulties of speech in noise.

It is important to consider several possible explanations for the absence of a relationship between the EFR and the performance on a speech-in-noise task. First, poorer speech-in-noise performance has often been associated with decreased robustness of F0 encoding (Anderson et al. 2011; Song et al. 2011). However, the present data suggest that aging does not necessarily lead to a reduction in the EFR spectral power at F0 (see also Anderson et al. 2012). Second, it has been argued that decreased speech encoding relevant for the perception of speech in noise particularly becomes apparent for stimuli with rapidly changing acoustic features (e.g., the formant transition period in /dɑ/; Hornickel et al. 2009; Song et al. 2011; Anderson et al. 2013). The present study used a steady-state vowel, however, in order to be able to assess the effects of amplitude fluctuations in the masker. Given the reduced robustness of the EFR in response to a steady-state vowel, it is reasonable to speculate that the older adults would also show decreased response properties for a rapidly changing stimulus. However, the fact remains that the older adults did not have increased difficulties understanding speech in SS or AM noise. It would therefore be unlikely that a potential group difference in the EFR to, for instance, a formant transition would relate to the older adults’ abilities to understand speech in noise. Lastly, it may simply be that the amount of disruption of neural speech coding was not severe enough to affect speech-in-noise performance. Speech perception, particularly in the presence of background noise, is a complex process that involves both top-down and bottom-up processes. It may thus be the case that older adults who show diminished precision of subcortical speech coding can somehow compensate for these degradations.

The data furthermore suggest that normal-hearing older adults do not experience reduced neural release from masking. While older adults show less robust EFRs in AM noise overall, they benefit just as much as younger listeners from amplitude dips in the maskers, with more robust response properties at the trough compared to the peak of the masker. This is perhaps not so surprising since behavioral measures and auditory steady-state responses have indicated that age-related declines in envelope processing only become apparent at higher modulation rates (primarily above about 100 Hz; e.g., Purcell et al. 2004; Grose et al. 2009). There was also no significant relationship between neural masking release and the ability to listen in the dips of the fluctuating noise. The lack of association here could simply be due to the fact that there was limited variability in either FMB or neural masking release across listeners.

To summarize:

- Aging, in the absence of hearing loss (audiometric thresholds ≤25 dB HL up to 4 kHz in both ears and 6 kHz in at least one ear), is associated with a decline in the robustness of subcortical speech encoding, both in quiet and noise.
- Normal-hearing older adults do not show a decrease in neural masking release, as indicated by the differences in the robustness of the EFR at the peak and trough of an AM masker, at slow masker modulation rates (10 Hz).
- Age-related declines in subcortical neural speech encoding do not necessarily lead to increased difficulties understanding speech in noise. Variability in SRTs was best explained by audiometric thresholds (pure-tone average across 0.5–4 kHz), not by the EFR in quiet or noise.
- Neural masking release as reflected in the EFR in AM noise does not predict dip listening over and above the click ABR, at least not at relatively slow masker modulation rates.

## Figures and Tables

**FIG. 9:**
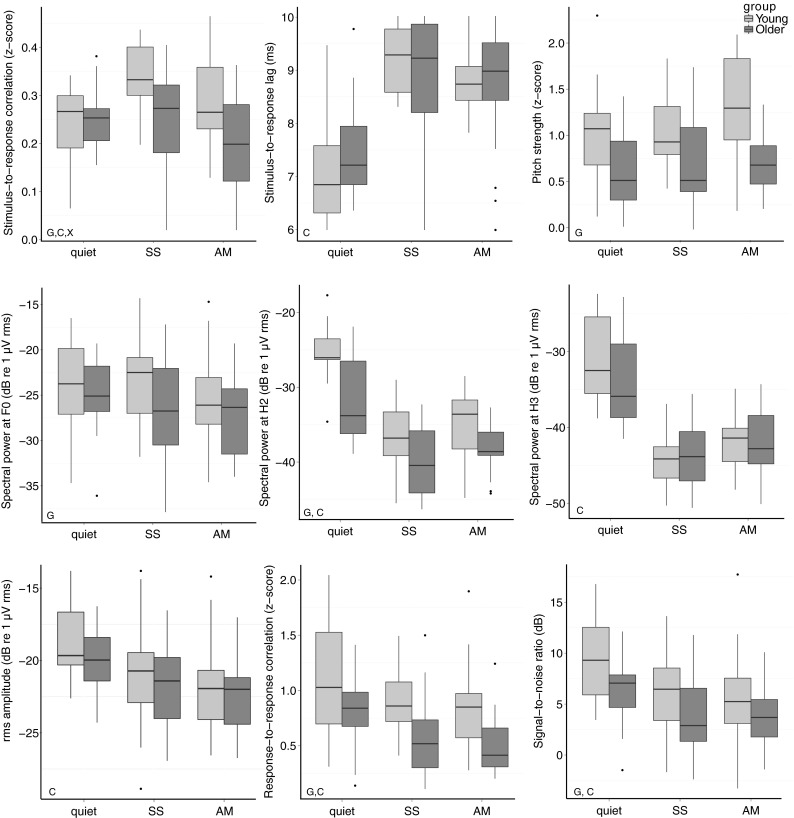
Boxplots of response properties of the EFR are shown for young (*light gray*) and older (*dark gray*) participants in the three different conditions (quiet, SS noise, and AM noise). The measures plotted here are the stimulus-to-response correlation (Fisher-transformed Pearson’s correlations, i.e., *z*-scores) (*top left*), stimulus-to-response lag (ms) (*top center*), pitch strength (z-score; *top right*), spectral power at the fundamental (F0) and second and third harmonics (dB re 1 μV rms; *mid-left*, *mid-center*, and *mid-right*, respectively), rms amplitude (dB re 1 μV rms) (*bottom left*), response-to-response correlations (Fisher-transformed Pearson’s correlations, i.e., z-scores; *bottom center*), and signal-to-noise ratio (SNR; *bottom right*). Any significant main effects of group (*G*) and condition (*C*), as well as any significant interactions (*X*), are indicated at the *bottom left-hand corner* of each panel. For details of the boxplot construction, see the caption to Fig. 4.

**FIG. 10:**
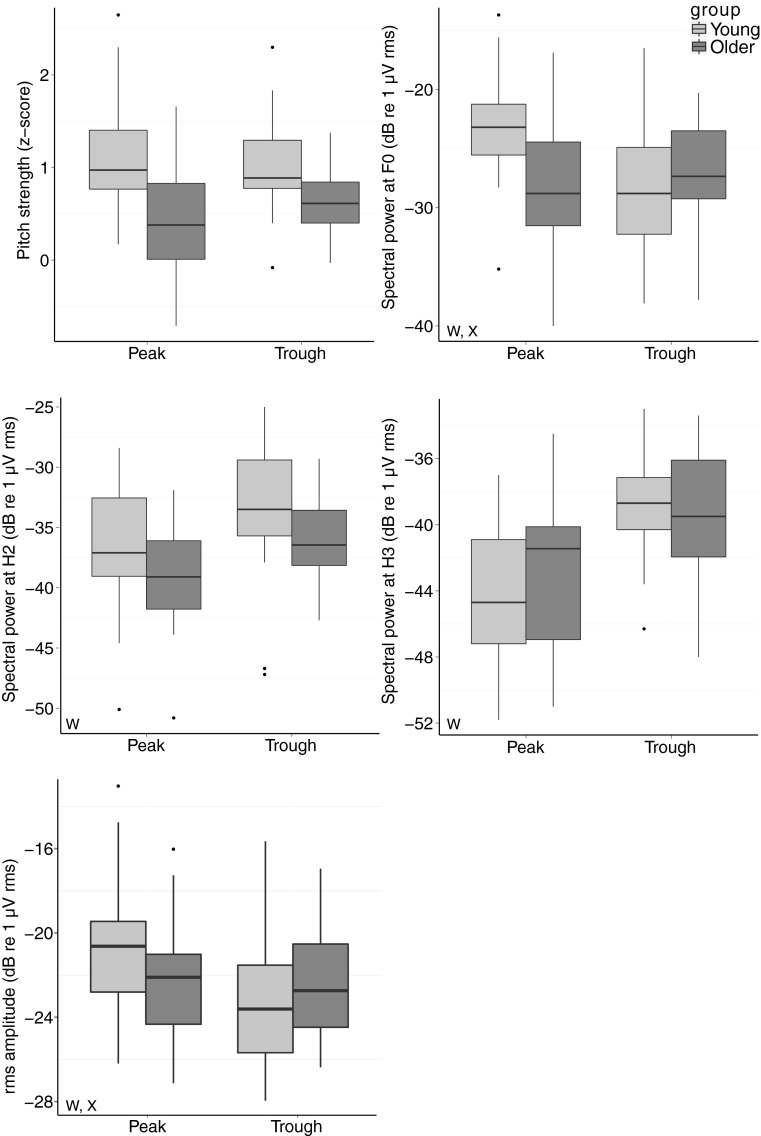
Boxplots of response properties of the EFR are shown for young (*light gray*) and older (*dark gray*) participants at the peak (*left*) and trough (*right*) of the AM noise. The measures plotted here are pitch strength (*top left*), spectral power at the fundamental (F0; dB re 1 μV rms; *top right*) and second and third harmonics (dB re 1 μV rms; *mid-left* and *mid-right*, respectively), and rms amplitude (dB re 1 μV rms; *bottom left*). Any significant effects of window (*W*) and any significant interactions (*X*) are indicated at the *bottom left-hand corner* of each panel. For details of the boxplot construction, see the caption to Fig. 4.

## Appendix

### Effects of Noise Maskers on the EFR

To assess the effects of noise on the individual EFR measures, several mixed-effects models were constructed. All models had condition (quiet, SS noise, and AM noise) and group (young, old) as fixed factors, and participant as a random factor. Since there was a large variation in the total number of sweeps included in the averaged EFR, models with “number of sweeps” as an additional continuous fixed factor were considered. Model comparisons based on the Akaike Information Criterion (AIC; Akaike 1974) indicated that a model with number of sweeps as an additional factor was a better fit for the data for seven out of eight outcome measures (see Table 5). Furthermore, given the difference in the sex balance between the two age groups (ten young compared to only three older males), models with sex as an additional fixed factor were also considered. Model comparisons suggested that incorporating sex as an additional factor was only a better fit when the outcome measure of interest was the spectral power at the second harmonic (see Table 5).

**TABLE 5:** Main effects of condition and group as well as interactions for the different EFR measures with significant effects highlighted in bold font (* significant at *α* = 0.05, ** significant at *α* = 0.01, *** significant at *α* = 0.001)

Mixed-effects models					
Measure	Condition	Group	Condition × group	Sweeps	Sex
Stimulus-to-response correlation	*F*_(2, 68)_ = 4.2	*F*_(1, 35)_ = 5.7	*F*_(2, 68)_ = 6.7		
	***p*** **= 0.02***	***p*** **= 0.02***	***p*** **= 0.002****		
Stimulus-to-response lag	*F*_(2, 60)_ = 15.6	*F*_(1, 35)_ = 0.2	*F*_(2, 60)_ = 2.2	*F*_(1, 60)_ = 0.2	
	***p*** **< 0.001*****	*p* = 0.7	*p* = 0.1	*p* = 0.6	
F0 strength	*F*_(2, 60)_ = 0.2	*F*_(1, 34)_ = 14.8	*F*_(2, 60)_ = 0.4	*F*_(1, 60)_ = 1.3	
	*p* = 0.7	***p*** **< 0.001*****	*p* = 0.7	*p* = 0.3	
Spectral power at F0	*F*_(2, 60)_ = 1.3	*F*_(1, 35)_ = 4.6	*F*_(2, 60)_ = 0.7	*F*_(1, 60)_ = 4.3	
	*p* = 0.3	***p*** **= 0.04***	*p* = 0.5	***p*** **= 0.04***	
Spectral power at H2	*F*_(2, 60)_ = 19.2	*F*_(1, 34)_ = 10.8	*F*_(2, 60)_ = 2.1	*F*_(1, 60)_ = 2.5	*F*_(1, 34)_ = 3.7
	***p*** **< 0.001*****	***p*** **= 0.002****	*p* = 0.13	*p* = 0.11	*p* = 0.06
Spectral power at H3	*F*_(2, 59)_ = 30	*F*_(1, 35)_ = 0.8	*F*_(2, 59)_ = 2.9	*F*_(1, 59)_ = 8.5	
	***p*** **< 0.001*****	*p* = 0.4	*p* = 0.06	***p*** **= 0.005****	
Rms amplitude	*F*_(2, 60)_ = 4.0	*F*_(1, 35)_ = 2.8	*F*_(2, 60)_ = 0.6	*F*_(1, 60)_ = 12.2	
	***p*** **= 0.02***	*p* = 0.1	*p* = 0.5	***p*** **< 0.001*****	
Response-to-response correlation	*F*_(2, 56)_ = 3.4	*F*_(1, 35)_ = 11.2	*F*_(2, 56)_ = 0.3	*F*_(1, 56)_ = 2.0	
	***p*** **= 0.04***	***p*** **= 0.002****	*p* = 0.7	*p* = 0.2	

The mixed-effects models for the different outcome measures differed in terms of the included fixed factors. Number of sweeps and sex were included as fixed factors when this improved the model fit

Overall, the results show that EFRs are degraded in the presence of background noise (both AM and SS). Furthermore, older adults had less robust responses compared to young adults. However, the EFRs of the older adults were typically not significantly more affected by background noise (i.e., no interactions, except in terms of the stimulus-to-response correlation). These results are illustrated in Figure 9.

The analyses indicated an interaction (condition × group) only for stimulus-to-response correlations. Post hoc independent *t* tests revealed a significant difference between the two age groups for AM and SS noise [AM: *t*_(35)_ = 2.5, *p* = 0.02, Cohen’s *d* = 0.55; SS: *t*_(30)_ = 2.9, *p* = 0.006, Cohen’s *d* = 0.65], with lower correlations for the older group. However, no significant group difference was found for the stimulus-to-response correlations for the EFRs in quiet [*t*_(33)_ = −0.3, *p* = 0.8]. This would suggest that aging primarily affects the robustness of subcortical encoding of speech in noise (and not quiet). Remember, however, that of the eight measures assessed, an interaction between condition and group was only found for stimulus-to-response correlation.

Four of the remaining seven outcome measures (i.e., not including the stimulus-to-response correlation) showed a significant main effect of group. The measures all followed the same pattern with less robust EFRs for the older adults. The EFRs of older adults are characterized by lower spectral power at the fundamental and the second harmonic, lower pitch strength, and lower response-to-response correlations. After Bonferroni correction, the group difference in spectral power at the fundamental is no longer significant.

Five out of seven outcome measures (i.e., not including the stimulus-to-response correlation) showed a significant main effect of condition, namely, the stimulus-to-response lag, spectral power at the second and third harmonics, rms amplitude, and response-to-response correlation. After Bonferroni correction, the condition effects remain significant for three measures (stimulus-to-response lag and the spectral power at the second and third harmonics).

Planned contrasts were carried out to further examine the effects of condition on the EFR. Two orthogonal contrasts were defined to examine (a) the overall effect of background noise on the EFR (quiet vs. AM and SS) and (b) the effect of amplitude modulation in the masker on the EFR more specifically (AM vs. SS).

The results of the planned contrasts are shown in Table 6. While the results overall indicated that EFRs were degraded in noise (quiet vs. AM and SS; all significant after Bonferroni correction), there was no difference between EFRs in AM and SS noise. The degrading effect of noise can be characterized by larger stimulus-to-response lags, lower spectral power at the second and third harmonics, lower rms amplitudes, and lower response-to-response correlations. After Bonferroni correction, the effect of background noise is no longer significant for the rms amplitude and the response-to-response correlation.

**TABLE 6:** Results from post hoc *t* tests for the EFR measures that showed a main effect of condition

Measure	Planned contrasts	
	Quiet vs. noise	AM vs. SS noise
Stimulus-to-response lag	*t*_(60)_ = −5.3	*t*_(60)_ = −0.2
	***p*** **< 0.001*****	*p* = 0.8
Spectral power at H2	*t*_(60)_ = 5.8	*t*_(60)_ = −1.8
	***p*** **< 0.001*****	*p* = 0.3
Spectral power at H3	*t*_(59)_ = 7.4	*t*_(59)_ = −0.8
	***p*** **< 0.001*****	*p* = 0.4
Rms amplitude	*t*_(60)_ = 2.5	*t*_(66)_ = 1.0
	***p*** **= 0.01***	*p* = 0.3
Response-to-response correlation	*t*_(56)_ = 2.3	*t*_(56)_ = 0.9
	***p*** **= 0.02***	*p* = 0.4

Significant effects are highlighted in bold font (* significant at *α* = 0.05, *** significant at *α* = 0.001)

### Effects of Amplitude Fluctuations in the Masker on the EFR

While the analyses described above did not reveal any differences between EFRs in SS and AM noise, this does not necessarily mean that amplitude fluctuations of the masker have no effect on the EFR. To examine the effects of amplitude fluctuations in the noise on the robustness of subcortical speech encoding, response measures of the EFR were compared across two analysis windows, one centered at the peak and one at the trough of the fluctuating masker. Mixed-effects models with window (peak, trough) and group (young, old) as fixed factors and participant as random factor were carried out for four response measures. The results are summarized in Table 7. Results for the main effect of group are not reported here. While the EFRs in AM noise may *overall* be less robust in the older compared to the younger adults (as seen in the analyses described above), only group effects in *relative differences* in the EFR measures between the peak and trough of the AM noise (i.e., interactions) are of interest here.

**TABLE 7:** Results from mixed-effects models examining the effects of amplitude modulations in the masker on the EFR. Results show the main effect of window (i.e., peak or trough in the AM masker) and the interaction between window and group for several EFR measures

Measure	Mixed-effects models	
	Window	Window × group
F0 strength	*F*_(1, 35)_ = 0.05	*F*_(1, 35)_ = 1.6
	*p* = 0.8	*p* = 0.2
Spectral power at F0	*F*_(1, 34)_ = 5.4	*F*_(1, 35)_ = 9.5
	***p*** **= 0.03***	***p*** **= 0.003****
Spectral power at H2	*F*_(1, 35)_ = 8.9	*F*_(1, 35)_ = 0.09
	***p*** **= 0.005****	*p* = 0.8
Spectral power at H3	*F*_(1, 35)_ = 36.2	*F*_(1, 35)_ = 1.2
	***p*** **< 0.001*****	*p* = 0.3
Rms amplitude	*F*_(1, 35)_ = 11.8	*F*_(1, 35)_ = 7.9
	***p*** **= 0.002****	***p*** **= 0.008****

Significant effects are highlighted in bold font (* significant at *α* = 0.05, ** significant at *α* = 0.01, *** significant at *α* = 0.001)

The analyses indicate an overall effect of window, although the pattern of the effect is inconsistent (see Fig. 10). It would be expected that the response properties are more robust at the trough than at the peak of the masker, similar to the way the EFR is more robust in quiet than in noise. However, while this is true for the spectral power of the second and third harmonics, which are higher (i.e., better) at the trough of the masker, the spectral power of the fundamental (not significant after Bonferroni correction) and the rms amplitude are in fact higher (i.e., better) at the peak of the masker. In addition, the EFR did not differ significantly across the two analysis windows in terms of pitch strength.

The fact that there are no interactions for the spectral power of the second and third harmonics suggests that both young and older listeners show a neural release from masking characterized by larger spectral power at the trough than at the peak of the AM noise. A closer examination of the interactions found for the spectral power at F0 and the rms amplitude indicates that these effects were mainly driven by the young adults. Post hoc paired *t* tests for these two measures revealed significant differences between the peak and trough of the masker only for the young participants [F0 young: *t*_(18)_ = 5.3, *p* < 0.001, Cohen’s *d* = 0.9; old: *t*_(17)_ = −0.5, *p* = 0.6; rms young: *t*_(18)_ = 4.6, *p* < 0.001, Cohen’s *d* = 0.7; old: *t*_(17)_ = 0.4, *p* = 0.7].
